# Novel Multistage Subunit *Mycobacterium tuberculosis* Nanoparticle Vaccine Confers Protection Against Experimental Infection in Prophylactic and Therapeutic Regimens

**DOI:** 10.3390/vaccines14010005

**Published:** 2025-12-19

**Authors:** Amir I. Tukhvatulin, Alina S. Dzharullaeva, Daria V. Vasina, Mikhail V. Fursov, Fatima M. Izhaeva, Denis A. Kleymenov, Dmitry N. Shcherbinin, Nikita B. Polyakov, Andrey I. Solovyev, Vladimir G. Zhukhovitsky, Alla S. Zhitkevich, Ilya V. Gordeychuk, Anna M. Litvinova, Victor A. Manuylov, Vasiliy D. Potapov, Artem P. Tkachuk, Vladimir A. Gushchin, Denis Y. Logunov, Alexander L. Gintsburg

**Affiliations:** 1 Federal State Budget Institution “National Research Centre for Epidemiology and Microbiology Named After Honorary Academician N. F. Gamaleya” of the Ministry of Health of the Russian Federation, 123098 Moscow, Russia; 2 The Federal Budgetary Institution of Science “State Research Center for Applied Microbiology and Biotechnology” of the Federal Service for Supervision of Consumer Rights Protection and Human Well-Being, Territory “Kvartal A”, 142279 Obolensk, Russia; 3 The Federal State Budgetary Educational Institution of Further Professional Education “Russian Medical Academy of Continuous Professional Education” of the Ministry of Healthcare of the Russian Federation, 125993 Moscow, Russia; 4 Chumakov Federal Scientific Center for Research and Development of Immune-and-Biological Products of Russian Academy of Sciences (Institute of Poliomyelitis), 108819 Moscow, Russia; 5 Department of Organization and Technology of Immunobiological Drugs, Sechenov First Moscow State Medical University, 119991 Moscow, Russia; 6 Department of Natural Sciences, Novosibirsk State University, 630090 Novosibirsk, Russia; 7 Federal State Budgetary Institution “National Medical Research Center for Phthisiopulmonology and Infectious Diseases” of the Ministry of Health of the Russian Federation, 127473 Moscow, Russia; 8 Department of Medical Genetics and Postgenomic Technologies, Sechenov First Moscow State Medical University, 119991 Moscow, Russia; 9 Department of Virology, Faculty of Biology, Lomonosov Moscow State University, 119234 Moscow, Russia; 10 Department of Infectology and Virology, Sechenov First Moscow State Medical University, 119991 Moscow, Russia

**Keywords:** *Mycobacterium tuberculosis*, TB, multi-stage vaccines, subunit vaccines, pattern-recognition receptors, PRR agonists

## Abstract

**Background/Objectives**: Tuberculosis (TB) remains the leading cause of death from a single infectious agent worldwide. In line with the World Health Organization’s (WHO) goal to end TB by 2035, the rapid development and clinical implementation of new, effective vaccines is urgently needed. To support global TB control efforts, we developed a novel candidate subunit multistage vaccine. **Methods:** This vaccine incorporates multiple *Mycobacterium tuberculosis* antigens expressed during both dormant and active stages of infection, fused into a single recombinant protein (ESAT6-CFP10-Ag85A-Rv2660c-Rv1813c). The antigen was encapsulated in biodegradable poly(D,L-lactide-co-glycolide) (PLGA) nanoparticles along with the pattern recognition receptor (PRR) agonists monophosphoryl lipid A (MPLA) and muramyl dipeptide (MDP), which function as adjuvants. **Results:** Using a mixed intramuscular/nasal prime-boost regimen, the vaccine elicited a mixed Th1/Th17 cell-mediated immune response, as well as a robust humoral response characterized by sustained systemic IgG (lasting at least one year) and prominent local secretory IgA. The vaccine demonstrated protective efficacy as a prophylactic booster following BCG priming in both murine and guinea pig models and was also effective in a therapeutic setting in a murine infection model. **Conclusions:** The results of this study provide empirical evidence that multistage tuberculosis vaccines represent a promising strategy for achieving global TB control.

## 1. Introduction

After the COVID-19 pandemic was no longer considered a global health emergency, tuberculosis (TB) regained its status as the leading cause of death from a single infectious agent [1]. According to a recent report, TB caused 1.25 million deaths in 2023 [2]. The high prevalence of TB—an estimated one-quarter of the world’s population has a latent infection—combined with the significant lifetime risk of developing active disease (approximately 5–10%), explains this substantial death toll. Since TB was declared a global health emergency in 1993, the World Health Organization (WHO) has aimed to end the global TB epidemic by 2035 [3]. However, the milestones and targets of the End TB Strategy are challenging to achieve. After a brief decline in diagnosed cases during the COVID-19 pandemic, largely due to disruptions in TB surveillance, the numbers have risen to the highest level since monitoring began—reaching 8.2 million in 2023 [4]. The epidemic is further complicated by drug-resistant TB. Although previously on a slow downward trend, the incidence of drug-resistant TB has remained stable since 2020 [5]. New TB vaccines, widely considered one of the most effective interventions against infectious diseases, are urgently needed to support the global health community’s efforts and to successfully implement the Global Plan to End TB.

Based on their administration regimens, tuberculosis (TB) vaccines can be divided into two main groups The first group, referred to as pre-exposure or prophylactic vaccines, is designed to generate protective immunity in individuals prior to *Mycobacterium tuberculosis* (*Mtb*) infection. This category includes the only licensed anti-TB vaccine for clinical use, Bacillus Calmette-Guérin (BCG), as well as numerous candidate vaccines designed to replace or supplement BCG as boosters [6,7]. The second group comprises vaccines that are administered in a therapeutic regimen to persons who have already been infected with *Mtb* [8]. Currently, a number of candidate vaccines based on different platforms (live-attenuated, whole cell inactivated, subunit, vectored) are also under intensive investigation in clinical trials [9]. In a stricter sense, ‘therapeutic’ vaccines administered to *Mtb*-infected individuals with the latent form of TB to prevent the manifestation of disease are referred to as ‘post-exposure’ vaccines. Whereas authentic ‘therapeutic’ vaccines are administered to persons who have already manifested symptoms of active disease in order to (i) increase the success rate of therapy (eradicate of *Mtb* as maximum), (ii) reduce the rate of disease recurrence, or (iii) shorten duration of antibiotic treatment or/and reduced number of drugs used. Alternatively, the TB vaccines can be classified according to the endpoint aim, which is the prevention of infection (POI), prevention of disease (POD) or prevention of recurrence (POR) [10]. However, developing new vaccines with strictly predefined roles based on such a classification may hinder the creation of truly effective measures for combating tuberculosis.

Apparently, during infection, *Mtb* may persist as a mixture of actively replicating and dormant forms, the proportions of which differ between different phases of the disease [11,12,13]. Given this, it is rational for new TB vaccines to deviate from established regimens and include antigens from multiple bacterial states. “Multistage” vaccines, which target antigens from various bacterial life stages (metabolically active and dormant states), represent a promising approach for inducing effective immune protection, potentially regardless of the vaccination strategy [14,15].

Following this logic, we prepared a novel candidate multi-antigenic vaccine based on fusion protein containing five *Mtb* antigens specific to active (Ag85A, ESAT6, CFP10) and latency-associated (Rv2660c, Rv1813c) stages of bacterial infection. These five antigens play distinct but critical roles in the pathogenesis of *Mycobacterium tuberculosis*. Ag85A (44 kDa) is a mycolyl-transferase essential for cell wall synthesis in both *Mtb* and BCG [16]. In contrast, ESAT-6 (6 kDa) and its chaperone-like partner CFP-10 (10 kDa) are absent from BCG strains; they form a 1:1 heterodimer that exhibits pore-forming activity, which is crucial for establishing *Mtb* infection [17]. Finally, the DosR regulon-encoded latency antigens Rv2660c (7.5 kDa) and Rv1813c (15 kDa) are crucial for the bacterium’s ability to enter and persist in a non-replicating state [18,19]. Because of their important roles in bacterial pathogenesis and their content of T-cell epitopes, these five antigens have become central targets for developing new tuberculosis vaccines and diagnostic assays [20]. The prepared ESAT6-CFP10-Ag85A-Rv2660c-Rv1813c fusion protein, combined with a highly active immunoadjuvant complex of TLR4 (MPLA) and NOD2 (MDP) agonists designed to mimic bacterial structure, was encapsulated into poly(D,L-lactide-co-glycolide) (PLGA) polymer nanoparticles.

Biodegradable polymers such as poly(lactic acid) (PLA), poly(glycolic acid) (PGA), and their copolymer PLGA are in high demand as subunit vaccine delivery systems [21]. PLGA, in particular, is one of the most extensively studied due to its approval by the FDA and EMA for pharmaceutical applications, its excellent safety profile, and its versatile delivery capabilities [22]. The mechanism by which PLGA nanoparticles enhance vaccine immunogenicity is multifaceted. First, with a typical size range of 100–500 nm, they mimic pathogens, facilitating efficient uptake by antigen-presenting cells (APCs) such as dendritic cells and macrophages [23,24]. Following internalization, PLGA nanoparticles enable antigen cross-presentation via both MHC class I and class II pathways, which is critical for inducing robust cellular and humoral immune responses. Additionally, the particles create a depot effect at the injection site, resulting in sustained antigen release over weeks to months. This prolongs antigen presentation and can eliminate the need for multiple booster injections. For example, one study demonstrated that a single dose of PLGA nanoparticles loaded with an Ag85B-ESAT6 fusion protein (H1) provided long-term protection in mice, matching the efficacy of multiple doses of the antigen alone [23]. Although PLGA nanoparticle vaccine formulations have shown promising results in various preclinical infection models—including tuberculosis, influenza, dengue, chlamydia, toxoplasmosis, varicella-zoster, and malaria—as well as in cancer vaccine platforms (particularly when combined with immune checkpoint blockade therapies), none has yet been approved for clinical vaccination. A few candidates, however, are currently in early-stage clinical trials. One example is PLGA nanoparticles loaded with a tumor antigen and an iNKT-cell agonist, now in phase 1 [25].

The present study demonstrates for the first time that a subunit PLGA-based vaccine containing five *Mtb* antigens with two different agonists of pattern-recognition receptors (PRRs) induces protection against tuberculosis infection in prophylactic and therapeutic regimens.

## 2. Materials and Methods

### 2.1. Primers Used for Genes Amplification

PCR was performed via C1000 Thermal Cycler (Bio-Rad, Hercules, CA, USA) using the ScreenMix-HS reagent kit (Evrogen, Moscow, Russia) and following forward (f) and reverse (r) primers:

ESAT6-f 5′-aagaaggagatatacatatgacagagcagcagtggaatttcg-3′,

ESAT6-r 5′-caccgccaccgctaccaccgccaccgctgccaccgccacctgcgaacatcccagtgacgttg-3′,

Cfp10-f 5′-cggtggtagcggtggcggtggcagcggtggcggtggttccatggcagagatgaagaccgatg-3′,

Cfp10-r 5′-agagcctgaaccactaccgctaccgaaccccatctgagaagac-3′,

Ag85a-f 5′-tagtggttcaggctctggctccggccagcttgttgacagggtt-3′,

Ag85a-r 5′-agaccctgatccacttccgcttccggcgccctgggg-3′

Rv2660c-f 5′-gcggaagtggatcagggtctgggtccggggtgatagcgggcgtcgaccagg-3′,

Rv2660c-r 5′-tccagaacctccgctgccaccgtgaaactggttcaatcccag-3′,

Rv1813c-f 5′-gtggcagcggaggttctggaggctcaggtgggcatctcgccaacggttc-3′

Rv1813c-r 5′-atggtgatggtggtgatggtggtgctcgaggttgcacgcccagttgac-3′.

The resulting sequence was checked for errors via Sanger sequencing using primers specific for vector pET28a part:

Vector-f 5′-tcaccaccatcaccattaaaagcttataaagattatggaagg-3′,

Vector-r 5′-catatgtatatctccttcttaaagtta-3′.

### 2.2. Fusion Protein Expression and Purification

The expression vector encoding the fusion protein was transformed into competent *E. coli* BL21 (DE3) cells (NEB, Ipswich, MA, USA) using a heat-shock protocol. The transformed cells were grown in LB medium at 37 °C with shaking at 240 rpm until the OD_600_ reached 0.55–0.65. Protein expression was then induced with 1 mM isopropyl β-D-1-thiogalactopyranoside (IPTG, Merck, Darmstadt, Germany) for 3 h at 37 °C.

The cells were harvested by centrifugation (6000× *g* for 10 min at 4 °C) and resuspended in lysis buffer (20 mM Tris-HCl, 0.1% Triton X-100, 1 mM PMSF, 5 mM EDTA, 5 mM β-mercaptoethanol, and 0.25 mg/mL lysozyme, pH 8.8; all from Merck, Rahway, NJ, USA). After centrifugation (12,000× *g* for 30 min at 4 °C), the pellet was washed three times with lysis buffer and frozen overnight.

The following day, the inclusion body-containing pellet was resuspended and subjected to sonication on ice for five 10 min cycles at 40% amplitude using a Branson 450 Sonifier (Emerson Electric Co., St. Louis, MO, USA). The sonicated material was solubilized in a denaturing buffer containing 8 M urea, 20 mM Tris, 0.15 M NaCl, 5 mM β-mercaptoethanol, 0.1% Triton X-100, and 40 mM imidazole (pH 8.8). The solution was then sterilized by filtration through a 0.22 µm PVDF filter (Merck, Darmstadt, Germany).

The fusion protein was purified under denaturing conditions using an ÄKTA pure system (Cytiva, Marlborough, MA, USA) equipped with a HisTrap HP column (Cytiva, USA). The filtered lysate was loaded onto the column, which had been pre-equilibrated with binding buffer (8 M urea, 20 mM Tris-HCl, 0.15 M NaCl, 5 mM β-mercaptoethanol, 0.1% Triton X-100, and 40 mM imidazole, pH 8.8). Bound proteins were eluted with a linear gradient to 100% elution buffer (8 M urea, 20 mM Tris-HCl, 0.15 M NaCl, and 500 mM imidazole, pH 8.0). The purified protein fractions were collected and dialyzed against 20 mM Tris-HCl (pH 8.8).

### 2.3. SDS-Page and Western Blotting Techniques

The concentration of the purified fusion protein was quantified using the Qubit Protein Broad Range (BR) Assay Kit (Thermo Fisher Scientific, Waltham, MA, USA). Its purity and identity were confirmed by SDS-PAGE, Western blot, and LC–MS/MS analyses. The reference proteins Ag85A and ESAT6 were purchased from Abcam (Cambridge, UK). The remaining proteins—CFP10, Rv1813c, and Rv2660c (the latter fused to a dextran-binding domain, DBD)—were expressed and purified using the same protocol as the fusion protein.

For SDS-PAGE, 5 µg of denatured protein in Laemmli buffer was resolved on 12% stain-free Mini-PROTEAN TGX gels (Bio-Rad, USA) at 100 V for 2 h. For immunoblotting, the proteins were transferred to a PVDF Hybond P membrane (Merck, USA) using a Trans-Blot SD Semi-Dry Transfer Cell (Bio-Rad, USA) at 20 V for 30 min.

The membranes were blocked overnight at +4 °C with stirring at 300 rpm in a solution of 5.0% skimmed milk and 0.05% Tween-20 in PBS (Merck, USA). The fusion antigen was detected using HRP-conjugated mouse anti-6xHis tag antibodies at a 1:50,000 dilution (Abcam, UK). Specific *Mtb* antigens were probed with the following: rabbit anti-Ag85A (1:30,000; Antibodies-online, Aachen, Germany), mouse anti-ESAT6 (1:5000; Abcam, UK), rabbit anti-CFP10 (1:10,000; Abcam, UK) monoclonal antibodies, as well as mouse anti-Rv1813c and anti-Rv2660c antisera (both at 1:5000 dilution) derived from animals immunized with the corresponding individual proteins.

Following incubation with the primary antibodies, the membranes were washed three times with TBS-Tween-20 buffer and then incubated for 1 h at 37 °C with shaking at 300 rpm with HRP-conjugated secondary antibodies: goat anti-rabbit (1:5000) and goat anti-mouse (1:4000) (both from Cytiva, USA). Finally, the membranes were washed three times with TBS-Tween-20 buffer, and the signal was developed using a Clarity Western ECL kit and visualized with a Gel Doc XR+ imager (both from Bio-Rad, USA).

### 2.4. High-Resolution LC–MS/MS Analysis of ESAT6-CFP10-Ag85A-Rv2660c-Rv1813c Fusion Protein

To confirm the primary structure of the recombinant fusion protein, an in-solution tryptic digestion was performed. The protein was dissolved in 100 mM ammonium bicarbonate (pH 8.3), reduced with tris(2-carboxyethyl)phosphine, and alkylated with chloroacetamide. Digestion was carried out at 37 °C for 12 h using trypsin at a 1:50 (*w*/*w*) enzyme-to-substrate ratio. The reaction was quenched with formic acid, and the resulting peptides were desalted and dried.

The peptides were separated using a nanoElute UHPLC system (Bruker Daltonics, Billerica, MA, USA) equipped with a C18 column and a 30 min linear gradient from 2% to 35% acetonitrile. Mass spectrometric analysis was performed on a timsTOF instrument (Bruker Daltonics, Germany) operated in positive ion, data-dependent acquisition mode.

Data were processed using PEAKS Studio software v11.0 (Bioinformatics Solutions Inc., Waterloo, ON, Canada). The spectra were searched against the theoretical protein sequence, an *E. coli* database, and a database of common contaminants. Search parameters included a parent mass tolerance of 20.0 ppm, a fragment mass tolerance of 0.02 Da, and semi-tryptic digestion allowing for up to three missed cleavages. Peptide and protein identifications were filtered to a 1% false discovery rate.

### 2.5. Preparation of Vaccine Nanoparticles

Nanoparticles (NPs) based on PLGA copolymer were prepared using a double emulsification–solvent evaporation technique. Briefly, 200 mg of 75:25 PLGA (Mw 66,000–107,000; Merck, Darmstadt, Germany) was dissolved in 10 mL of ethyl acetate (Merck, USA) as the organic solvent in a 50 mL centrifuge tube (Corning, New York, NY, USA). The tube was sonicated in an ultrasonic bath for 30 min to achieve complete polymer dissolution.

Separately, the primary aqueous phase was prepared by dissolving 2.5 mg of fusion protein in 1 mL of phosphate-buffered saline (PBS) containing the immunostimulatory molecules MPLA (0.35 mg) and MDP (1.25 mg) (all from Invivogen, San Diego, CA, USA). This aqueous phase was added dropwise to the PLGA solution and then sonicated on ice using a Branson 450 Sonifier at 30 W for 30 s (15 cycles of 2 s on and 2 s off) to form the primary water-in-oil (*w*/*o*) emulsion.

A secondary aqueous phase, consisting of 40 mL of a 1% (*w*/*v*) aqueous solution of 9–10 kDa poly(vinyl alcohol) (PVA, Merck, USA), was then added to the primary emulsion. The mixture was processed using an LM20 Microfluidizer (Microfluidics, Westwood, MA, USA) for 5 cycles at 10,000 psi to form a nanoscale double emulsion (*w*/*o*/*w*). The final emulsion was stirred magnetically for 4 h at 37 °C to allow for ethyl acetate evaporation.

The resulting nanoparticles were collected and washed with 2 L of deionized water using an Acta Flux S tangential flow filtration system equipped with a 750 kDa hollow fiber filter (both from Cytiva, USA). The washed nanoparticulate suspension was lyophilized with 0.1% (*w*/*v*) sucrose as a cryoprotectant using a 2.5 L Freezone Plus Freeze Dry System (Labconco, Kansas City, MO, USA). The final PLGA NP samples were sealed aseptically and stored at −20 °C until use.

### 2.6. Characterization of Physical Parameters of Vaccine NPs

The average particle size, polydispersity index (PDI), and zeta potential were measured using a Malvern Zetasizer Nano instrument (Malvern Instruments, Worcestershire, UK) and analyzed with Zetasizer 7.01 software. The average particle size and PDI were determined by dynamic light scattering (DLS) after diluting the vaccine to 100 µg/mL in 10 mM Tris buffer (pH 7.4) using a UV microcuvette (BrandTech, New York, NY, USA). The zeta potential was measured by laser Doppler electrophoresis following dilution of the vaccine nanoparticles (NPs) to 1 µg/mL in 10 mM Tris buffer (pH 7.4) using folded capillary cells (Malvern Instruments, UK).

The average particle concentration and size distribution were estimated by nanoparticle tracking analysis (NTA) using a NanoSight NS300 system (Malvern Technologies, UK) equipped with a 488 nm laser and a CMOS camera. The vaccine NPs were diluted to 0.01 µg/mL in particle-free PBS, which had been filtered through a 0.02 µm membrane (Whatman, UK). Samples were analyzed under constant flow conditions at a flow rate of 60 µL/min and a temperature of 25 °C. Data were processed using NTA 3.2 software (Malvern Instruments, UK). To ensure consistent calculation of particle distribution and concentration, three 60 s videos were captured for each sample with the camera level set between 9 and 14 and a detection threshold of 4.

For transmission electron microscopy (TEM) analysis, particles were adsorbed for 5 min onto copper grids coated with carbon/formvar (Formvar/Carbon 200 Mesh, carbon 3–4 nm, FCF200-CU-SB, Electron Microscopy Sciences, Hatfield, PA, USA). The grids were then washed seven times with 1 M EDTA (PanReac AppliChem, Barcelona, Spain), negatively stained with 2% uranyl acetate (Serva, Heidelberg, Germany) for 2 min, air-dried, and examined using a JEM-100C transmission electron microscope (Jeol, Tokyo, Japan) at a magnification of 60,000×. The size of the particles was determined using the BioVision 4.0 program (West Medica, Wiener Neudorf, Austria).

### 2.7. Measurement of Antigen Encapsulated in Vaccine NPs

First, vaccine nanoparticles (NPs; 6 mg per sample) were lysed in 1 mL of 0.1 M NaOH by sonication for 30 min using an ultrasonic bath (Biosan, Riga, Latvia). The pH was then neutralized with 0.2 M HCl before measuring the antigen encapsulation efficiency. The protein content in the lysates was quantified with a Bicinchoninic Acid Protein Assay Kit (PanReac AppliChem, Barcelona, Spain) according to the manufacturer’s instructions. Briefly, 150 μL of each sample was added to a 96-well plate along with 75 μL of Reagent A, 72 μL of Reagent B, and 3 μL of Reagent C. To determine the protein concentration, a calibration curve was generated using 150 μL aliquots of the fusion protein at concentrations ranging from 1 to 250 μg/mL. The plate was incubated at 37 °C for 30 min, and the absorbance was measured at 562 nm for all samples using a Synergy H1 microplate reader (Biotek, Winooski, VT, USA). Encapsulation efficiency (EE) for the protein or PRR agonists was calculated according to the formula: EE (%) = (Amount of antigen encapsulated in NPs/Total antigen amount used) × 100. The loading capacity (LC) of the PLGA nanoparticles was determined as: LC (%) = (Amount of antigen in lyophilized NPs/Total mass of lyophilized NPs) × 100. The achieved EE and LC were not maximized, as the study priorities were (1) generating uniformly sized nanoparticles (100–200 nm) and (2) optimizing the depot effect of the PLGA carrier

### 2.8. Measurement of PRR Agonists Internalized in Vaccine NPs

Vaccine NPs were lysed and neutralized as described above. The processed samples were then added to HEK-Blue-hTLR4 and HEK-Blue-NOD2 reporter cells (all from Invivogen, USA). These cells stably express the human TLR4 or NOD2 receptors, along with an NF-κB/AP-1-inducible secreted embryonic alkaline phosphatase (SEAP) reporter gene. Soluble MPLA and MDP were used as standards to generate a calibration curve, and parental HEK-Blue-Null2 cells served as a negative control.

Reporter cells were seeded in 96-well plates at a density of 2 × 10^4^ cells per well in complete DMEM medium. The following day, the cells were treated with the processed vaccine NP samples or soluble pattern recognition receptor (PRR) agonists at the indicated concentrations. After an 18 h incubation, SEAP activity was quantified by mixing 50 µL of clarified culture supernatant with 150 µL of 60 µM p-nitrophenylphosphate (Merck, Germany) in SEAP assay buffer (0.5 M carbonate, pH 9.8, 0.5 mM MgCl_2_). Absorbance was measured at 405 nm using a Synergy H1 microplate reader (Biotek, USA).

### 2.9. Animal Studies

Immunogenicity studies were conducted at the N.F. Gamaleya National Research Center for Epidemiology and Microbiology. Female C57BL/6 specific-pathogen-free (SPF) mice (4–5 weeks old) were obtained from the center’s on-site breeding facility. The mice were housed in ventilated ISOCage P systems (Techniplast, Buguggiate, Italy) with free access to autoclaved water and a standard diet. They received two intramuscular (IM) injections into the thigh muscle, followed by one intranasal (IN) administration of the vaccine nanoparticles (NPs), with a two-week interval between each immunization. For IN administration, the mice were anesthetized with isoflurane (Abbott, Chicago, IL, USA).

The vaccine’s protective efficacy was assessed at the State Research Center for Applied Microbiology and Biotechnology (Obolensk, Russia). Female C57BL/6 mice and albino guinea pigs (7–8 weeks old) were purchased from the Scientific Center for Biomedical Technologies (Andreevka, Russia) and housed under SPF conditions in individually ventilated cages with ad libitum access to food and water. *Mycobacterium tuberculosis* (*Mtb*) H37Rv challenge experiments were conducted in an Animal Biosafety Level 3 (ABSL-3) laboratory at the State Research Center. In the pre-exposure prophylaxis model, C57BL/6 mice and albino guinea pigs were placed in an Inhalation Exposure System (Glas-Col, Terre Haute, IN, USA) and infected with *Mtb* H37Rv at a dose of 50–100 colony-forming units (CFU) per animal. In the post-exposure model, mice were infected intraperitoneally with a dose of 1 × 10^4^ CFU per mouse.

### 2.10. Sample Acquisition

The magnitude of the post-vaccination immune response was evaluated on day 42. At the experimental endpoint, mice were euthanized with an overdose of inhaled CO_2_. Blood and bronchoalveolar lavage (BAL) fluid were collected to assess the humoral immune response, while the spleen and regional (inguinal) lymph nodes were harvested to evaluate the cellular immune response.

The spleen and lymph nodes were minced using sterile scissors and pressed through a 40 µm nylon cell strainer (SPL, Pocheon-si, Republic of Korea) to obtain a single-cell suspension for further analysis. Blood was collected via cardiac puncture using a 1 mL syringe. Serum was isolated by incubating the blood at 37 °C for 30 min, followed by centrifugation at 800× *g* for 10 min.

BAL specimens were obtained by tracheal puncture with a 23-gauge needle catheter. A 0.5 mL volume of PBS was infused into the lungs and then aspirated. The aspirate was transferred to 1.7 mL tubes, placed on ice, and sonicated for 20 min in an ultrasonic water bath (ELMA, Wetzikon, Switzerland). The samples were then clarified by centrifugation at 1800 rpm and 4 °C for 10 min, immediately aliquoted, and stored at −80 °C until analysis.

To evaluate IgG kinetics after vaccination, serial blood collections (50 µL each) were performed via submandibular vein puncture from the same mice on days 42, 180, and 365.

### 2.11. Mycobacterium Strains

*Mtb* strain H37Rv and *M. tuberculosis* variant *bovis* (BCG) were obtained from the State Collection of Pathogenic Microorganisms “SCPM-Obolensk” (strains IDs B-4825 and B-4824, respectively). The bacteria were grown in Middlebrook 7H9 broth supplemented with OADC (BD, Franklin Lakes, NJ, USA) and 0.05% Tween-80 until the late log phase. Bacterial suspensions (1 × 10^7^ CFU/mL) were frozen and stored at −70 °C. Serial dilutions of the bacterial suspensions were plated on Middlebrook 7H10 or 7H11 agar plates for colony-forming unit (CFU) counts before use.

### 2.12. Analysis of Lymphoproliferative Response

Splenocytes (leukocytes from the spleen) were purified by density gradient centrifugation (400× *g* for 30 min) using Ficoll (1.09 g/mL; PanEco, Russia). Viable cell counts were determined by trypan blue exclusion using a TC-20 cell counter (Bio-Rad, USA). Splenocytes from each mouse (10^7^ cells) were stained with carboxyfluorescein diacetate succinimidyl ester (CFSE; Invitrogen, Waltham, MA, USA), seeded into 96-well plates at 2 × 10^5^ cells per well, and restimulated with either a fusion protein or individual *Mtb* antigens (1 µg/well). After 72 h, the cells were harvested, washed with PBS (400× *g* for 10 min), and stained for 20 min at 4 °C in the dark with DAPI (1 µg/mL) and the following antibodies in staining buffer: anti-CD3 PE-Cy7 (clone 145-2C11), anti-CD8 APC (clone 53–6.7), and anti-CD4 PE (clone RM4-5) (all from BD Biosciences, USA). Proliferation of CD4+ or CD8+ T lymphocytes was defined as the percentage of cells in the final culture that had undergone at least one division, corresponding to the ‘Fraction Divided’ statistic [18]. All samples were analyzed on a FACSAria III flow cytometer, and data were processed using FACSDiva and FlowJo v10.5.3. software (BD Biosciences, USA).

### 2.13. Cytokine Analysis

Splenocytes and cells from lymph nodes were seeded in 96-well plates at 2 × 10^5^ cells per well and restimulated with fusion protein (1 µg/well). Seventy-two hours later, cell-free culture supernatant samples were obtained for further cytokine analysis using the mouse 23-plex Th1/2 and 7-plex Th17 bead-based Bio-Plex Pro Kit (Bio-Rad Laboratories, USA) according to the manufacturer’s instructions.

### 2.14. Serum IgG Titers Measured by Bead-Based Assay

Antibody titers in the total IgG fraction from immunized mouse serum, specific to a fusion protein as well as to individual *Mtb* antigens (Ag85A, ESAT6, CFP10, Rv2660c, Rv1813c), were evaluated using a bead-based multiplex assay with xMAP technology (Luminex, Austin, TX, USA), as previously described [26].

Briefly, antigens were coupled to microspheres (5–20 µg per 10^6^ microspheres) according to established protocols [27,28]. For the assay, a mixture of 80 µL of CBS-T buffer (PBS, 1% BSA, 0.1% Tween-20, 0.05% NaN_3_), containing 2500 microspheres per antigen region, and 20 µL of serum (prediluted 20-fold with PVXC buffer; PBS, 0.8% polyvinylpyrrolidone, 0.1% casein, 0.5% PVA, 0.05% NaN_3_) was incubated for 60 min at 25 °C with shaking at 800 rpm in a Microlon 96 W microplate (Greiner, Kremsmünster, Austria).The microplates were then washed with PBS-TBN buffer (PBS, 0.1% BSA, 0.03% Tween-20, 0.05% NaN_3_) using a 405 TS Microplate Washer (BioTek, USA). Subsequently, 100 µL of a 2.5 µg/mL solution of phycoerythrin-conjugated anti-mouse IgG (ThermoFisher Scientific, MA, USA) was added to each well. A second incubation was performed for 30 min at 25 °C and 800 rpm, followed by another wash step. Finally, the microplates were analyzed using a MAGPIX system (Luminex, TX, USA). Vaccine-specific IgG antibodies were detected by measuring the mean fluorescence intensity (MFI) at each time point.

### 2.15. Serum and BAL Antibody Titers Measured by ELISA

First, 96-well plates (SPL, Republic of Korea) were coated with fusion protein (1 μg per well) in coating buffer (137 mM NaCl, 2.7 mM KCl, 8.1 mM Na2HPO4, and 1.5 mM KH2PO4) and incubated overnight at 4 °C. On the next day, plates were washed three times with PBS containing 0.05% Tween20 (PBS-T) and blocked with 3% nonfat milk in PBS-T (all, Merck, USA) for 1 h at 37 °C. Serum and BAL samples were added in 2-fold dilutions in blocking buffer, incubated 1 h at 37 °C, washed three times with PBS-T. Secondary goat anti-mouse antibodies (Abcam, UK) diluted 1:5000 in PBS-T were used for the detection of IgG and IgA antibodies. A colorimetric signal was detected after the addition of TMB peroxidase substrate (Merck, USA), stopped with 4 M H_2_SO_4_ and measured at 450 nm using a Synergy H1 reader (Biotek, USA).

### 2.16. Quantification of Bacterial Burden

To determine bacterial load, mice were euthanized by CO_2_ asphyxiation at designated time points. The lungs and spleens were aseptically harvested, homogenized in sterile PBS, and serially diluted ten-fold. Dilutions were plated on Middlebrook 7H11 agar supplemented with OADC and incubated at 37 °C for 21–28 days for colony enumeration.

### 2.17. Statistical Analysis

Statistical analysis was performed using GraphPad Prism 10.2. Data between two groups within one time point were compared using non-parametric Mann–Whitney U test. Time-dependent changes in registered parameters between experimental groups were analyzed using two-way ANOVA coupled with Tukey’s multiple-comparisons test.

## 3. Results

### 3.1. Preparation of Recombinant Fusion ESAT6-CFP10-Ag85A-Rv2660c-Rv1813c Protein

**Figure 1:**
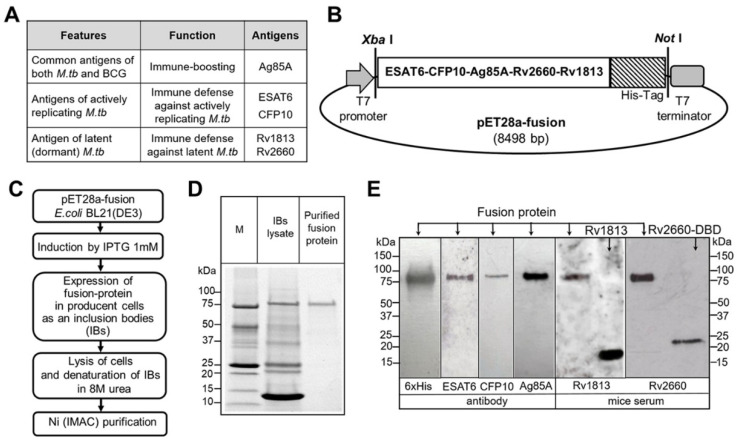
The production, purification, and identification of the recombinant fusion protein ESAT6-CFP10-Ag85A-Rv2660c-Rv1813c. (**A**) *Mtb* proteins selected for the fusion vaccine antigen, with brief descriptions. (**B**) The fusion gene encoding ESAT6-CFP10-Ag85A-Rv2660-Rv1813c with an N-terminal His-Tag sequence was cloned into a kanamycin-resistant pET28a expression vector for protein expression. (**C**) Schematic of protein expression and purification. The process involved the following steps: transformation of *E. coli* BL21 (DE3) with the pET28a-fusion plasmid; induction of fusion protein expression with 1 mM IPTG; harvesting of transformed bacteria at an OD620 > 2.5; isolation of the fusion protein from inclusion bodies (IBs); and final protein purification using immobilized metal (Ni2+) affinity chromatography (IMAC). (**D**) SDS-PAGE analysis of the expressed ESAT6-CFP10-Ag85A-Rv2660c-Rv1813c fusion protein in inclusion bodies and after chromatographic purification. M, protein marker. (**E**) Identification of *Mtb* proteins within the purified recombinant fusion antigen by Western blot analysis. The membrane with the transferred fusion protein was probed with commercial anti-ESAT6, anti-CFP10, anti-Ag85, or anti-6x-His-tag antibodies, as well as with mouse anti-Rv1813c or anti-Rv2660c serum. Hyperimmune sera were generated by immunizing Balb/c mice twice with recombinant Rv1813c or Rv2660c proteins. Rv1813c and a fusion Rv2660c protein with a dextran-binding domain (DBD) were loaded in separate lanes (as indicated) to confirm the specificity of the hyperimmune sera.

The nucleotide sequences of five *M. tuberculosis* (*Mtb*) antigens were PCR-amplified from the H37Rv strain. These antigens were selected for three purposes: (i) to boost the BCG vaccine (Ag85A), (ii) to mount immune protection against active bacilli (ESAT6, CFP10), and (iii) to target dormant bacilli (Rv2660c, Rv1813c) (Figure 1A).

The PCR products were cloned into the pET28a expression vector to create a single fusion gene. The individual antigen sequences were separated by Gly Ser spacers, and the construct included a C-terminal 8xHis tag (Figure 1B). Following induction with IPTG in *E. coli* BL21(DE3) cells, the resulting fusion protein was expressed in inclusion bodies. The protein was then purified under denaturing conditions using immobilized metal affinity chromatography (IMAC) with a Ni^2+^ resin (Figure 1C). SDS-PAGE analysis verified the protein’s purity, showing a single major band at approximately 80 kDa, which is consistent with its calculated molecular weight (Figure 1D). A Western blot analysis using anti-6xHis antibodies confirmed the identity of this band (Figure 1E). The presence of the specific *Mtb* antigens ESAT6, CFP10, and Ag85A within the fusion was confirmed by Western blot using commercial antibodies. Since commercial antibodies against Rv2660c and Rv1813c were unavailable, the presence of these antigens was detected using hyperimmune mouse serum from mice vaccinated with the individual recombinant proteins. Finally, the fusion protein sequence was verified by LC–MS/MS analysis (Supplementary Figure S1).

### 3.2. Preparation and Characterization of PLGA-Based Multistage Vaccine

**Figure 2:**
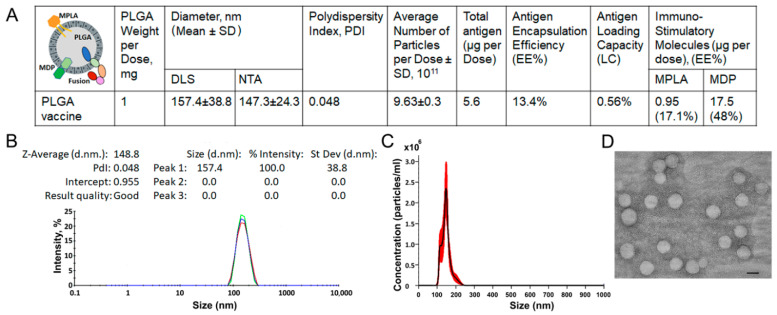
Characterization of PLGA vaccine nanoparticles. (**A**) Physico-chemical parameters and composition of PLGA-based multistage vaccine. Size distribution analysis by (**B**) dynamic light scattering (DLS) and (**C**) nanoparticle tracking analysis (NTA). (**D**) Transmission electron microscopy (TEM) image of the vaccine particles [scale bar: 100 nm Colors (red, blue, and green) represent results from three independent samples.

In the context of subunit vaccine design, the choice of adjuvant molecules plays a critical role in the overall immunogenicity and protective efficacy of a vaccine. To this end, we used a combination of MPLA (a TLR4 agonist) and MDP (an NOD2 agonist) as a complex immunoadjuvant, which has been shown to be highly effective when co-administered with various vaccine antigens [24,29]. To co-localize both the antigen and adjuvant molecules within vaccine particles, we used the clinically approved, non-toxic PLGA used as a carrier [30]. PLGA-based vaccine nanoparticles (NPs) were prepared using a double-emulsion solvent evaporation method. In this process, a high-shear homogenizer was used instead of sonication to achieve a finer and more monodisperse vaccine formulation. The vaccine preparation was characterized by key physicochemical parameters known to influence immunogenicity, including size, polydispersity, particle count, and the content of antigen and immunostimulatory molecules (Figure 2A). Particle size analysis by DLS and NTA showed a consistent mean size of approximately 150 nm (Figure 2B,C), which was confirmed by TEM, revealing spherical, homogeneous, and electron-dense particles of similar diameter (Figure 2D).

PLGA vaccine particles remained stable in 1 × PBS at room temperature for up to 28 days, as measured by their size, particle count, and antigen content (Supplementary Figure S2). These results align with previously published data indicating that a lower glycolide content, and thus a predominance of less hydrophilic lactide units, leads to slower degradation kinetics [31].

### 3.3. A PLGA-Based Multistage Vaccine Elicits a Robust T Cell-Mediated Immune Response Characterized by Th1/Th17 Polarity in Mice

**Figure 3:**
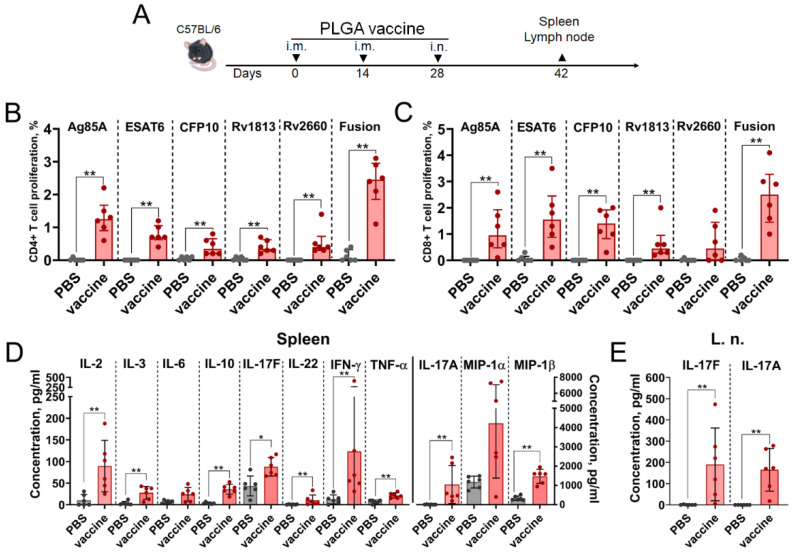
PLGA-based multistage vaccine drives a Th1/Th17-polarized T-cell response in mice. (**A**) C57BL/6 mice (6 per group) received three PLGA vaccinations (two intramuscular followed by intranasal) with 2-week interval. The placebo group received PBS on the same days. On day 42, spleens and regional lymph nodes (l.n.) were collected and isolated lymphocytes were seeded into 96-well plates. Percentages of proliferating (**B**) CD4+ and (**C**) CD8+ T cells from the spleen after restimulation with individual *Mtb* proteins (ESAT6, CFP10, Ag85A, Rv2660c, Rv1813c) or the fused vaccine antigen. Cytokine levels in cell-free culture supernatants of lymphocytes from the (**D**) spleen or (**E**) regional lymph nodes after restimulation with the fused vaccine antigen. Data points represent individual mice. Bars indicate the mean, and whiskers represent SD. Significant differences between vaccinated and non-vaccinated mice are indicated by asterisks (Mann–Whitney U-test, * *p* < 0.05, ** *p* < 0.01).

Adaptive cell-mediated immunity plays a pivotal role in resistance to *Mtb* infection. Combining intramuscular and intranasal administration routes in a vaccination regimen is a promising approach for achieving more potent anti-*Mtb* immune responses [32,33]. In this study, mice received two intramuscular injections of a prepared subunit PLGA-based vaccine, followed by one intranasal administration at two-week intervals (Figure 3A).

On day 42, spleens and regional (inguinal) lymph nodes were collected. Splenocytes were cultured to evaluate CD4+ and CD8+ T-cell lymphoproliferative responses after antigen restimulation using isolated *Mtb* antigens, as well as the whole fusion protein (Figure 3B,C). Stimulation with all five *Mtb* antigens triggered lymphoproliferative response of CD4+ and CD8 T cells; however, the percentages and proportions of proliferating CD4+ and CD8+ T-cells differed among them. The highest CD4+ and CD8+ proliferative responses of splenocytes (mean values of 2.35% and 2.45%, respectively) were detected following the addition of the fusion protein containing all five antigens. We also characterized the cytokine profiles secreted into the culture medium by immune cells isolated from spleens and regional (inguinal) lymph nodes after antigen restimulation. Due to low cell numbers in the lymph nodes, only the fusion protein was used for stimulation. In splenocyte cultures, we detected a statistically significant elevation of 11 out of 30 measured cytokines, which belonged to a mixed Th1 (IL-2, IL-10, TNFα, IFNɣ)/Th17 (IL-17A, IL-17F, IL-22) mediated immune response (Figure 3D). Interestingly, immune cells from the lymph nodes responded only by producing IL-17A and IL-17F, but at levels two-fold higher than those from splenic cells (Figure 3E).

### 3.4. A PLGA-Based Multistage Vaccine Induces Robust Humoral Immune Response in Mice

**Figure 4:**
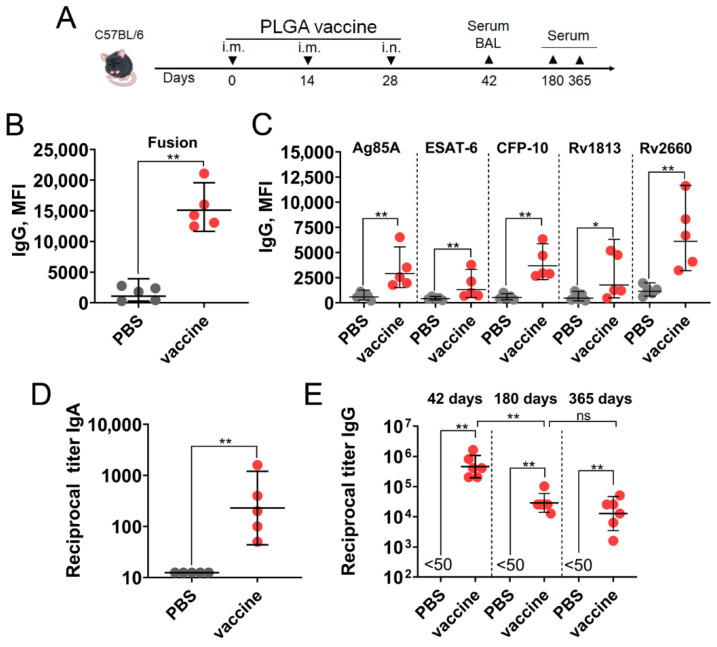
The PLGA-based multistage vaccine induces a robust humoral immune response in mice. (**A**) C57BL/6 mice (*n* = 5 per group) were immunized three times at two-week intervals. The regimen consisted of two intramuscular (i.m.) injections followed by one intranasal (i.n.) administration. The placebo group received PBS on the same schedule. Serum and bronchoalveolar lavage (BAL) fluid were collected on the indicated days to assess the magnitude of the humoral immune response. On day 42, antibody levels were evaluated by measuring (**B**) IgG against the whole fusion protein and (**C**) IgG against individual *Mtb* proteins in serum samples, as well as (**D**) IgA against the whole fusion protein in BAL samples. (**E**) Kinetics of endpoint IgG titers against the whole fusion protein in mouse serum. Dots represent individual data points. Horizontal lines represent the geometric mean, and whiskers indicate 95% confidence intervals (CIs). Significant differences between groups are indicated by asterisks (two-way ANOVA with Tukey’s multiple comparisons test; * *p* < 0.05, ** *p* < 0.01). ns, not significant.

Although *Mtb* is primarily an intracellular pathogen, its extracellular phase appears to be vulnerable to antibody-mediated immune mechanisms. Experimental studies show that antibodies targeting specific *Mtb* antigens can reduce bacterial loads and modulate inflammatory responses, suggesting a protective role for humoral immunity in TB [34]. To assess the humoral response, mice were immunized as described above. Serum and bronchoalveolar lavage (BAL) fluid were collected two weeks after the last immunization to measure levels of antigen-specific IgG and IgA titers, respectively (Figure 4A).

Using a bead-based assay, we detected prominent production of IgG antibodies against the full fusion protein and each of the five individual *Mtb* antigens (Figure 4B,C). The mixed intramuscular/intranasal immunization also induced effective formation of anti-fusion IgA antibodies, which were detected in the bronchoalveolar lavage fluid (Figure 4D). To evaluate the duration of the vaccine-mediated humoral immune response, we measured end-point titers of fusion-specific IgG antibodies at distant time points (days 180 and 365) and compared them to the titer at day 42. We found that although the geometric mean reciprocal titer (GMRT) of IgG antibodies significantly declined from its peak at day 42 (459,760), it remained at substantial levels six months (28,735) and one year (12,800) after vaccination (Figure 4E).

### 3.5. PLGA-Based Multistage Vaccine Enhances BCG-Mediated Protection in Pre-Exposure Infection Model

**Figure 5:**
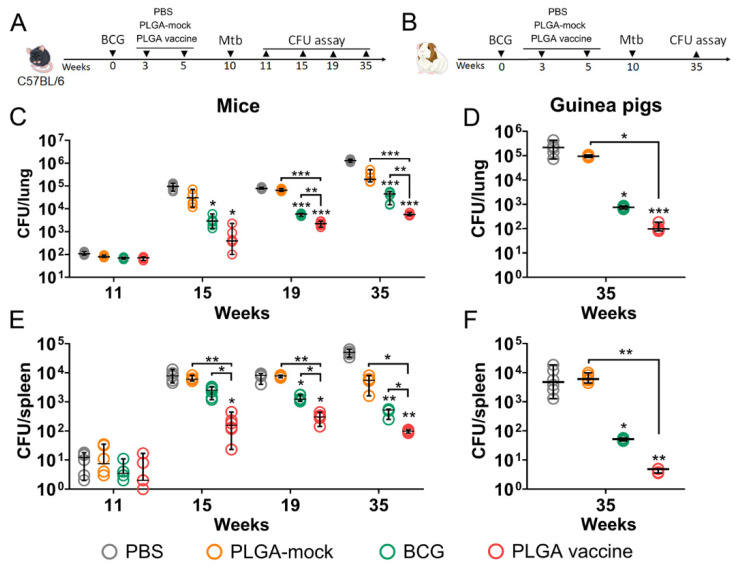
The protective efficacy of the PLGA-based multistage vaccine used in prophylactic regimen as a booster for BCG primary immunization. (**A**) C57BL/6 mice and (**B**) guinea pigs were immunized with BCG vaccine (5 × 10^6^ CFU/mouse). 3 weeks later animals were randomly split to experimental groups and boosted with placebo (BCG), PLGA particles without fusion antigen (PLGA-mock) or PLGA-vaccine in 2-dose immunization scheme with 2 weeks interval. Control animals received PBS at each vaccination (PBS). 5 weeks after the last vaccination animals were challenged with aerosolized *M. tuberculosis* H37Rv at 100 CFU/animal. Bacterial loads in mouse (**C**) lungs and (**D**) spleens were assessed at week 11, 15, 19 and 35 (6 animals per group). Bacterial loads in guinea pigs’ (**E**) lungs and (**F**) spleens were assessed at week 35 (6 animals per group). Dots represent individual data. Horizontal lines indicate the mean, and whiskers represent SD. Significant differences between different experimental groups are indicated by asterisks (two-way ANOVA coupled with Tukey’s multiple-comparisons test, * *p* < 0.05, ** *p* < 0.01, *** *p* < 0.005).

Given that worldwide BCG coverage exceeds 80%, we employed a pre-exposure regimen using a PLGA vaccine as a booster following primary BCG vaccination [35]. The vaccine’s protective efficacy was assessed in mice and guinea pigs using a prime-boost schedule. The first immunization was performed with the BCG vaccine intramuscularly, followed by a second (intramuscular) and a third (intranasal) booster with the PLGA vaccine (Figure 5A,B). Control groups included unvaccinated animals (PBS group), animals primed with BCG that received PLGA particles containing only immunostimulatory molecules (PLGA-mock group), and BCG-primed animals that were left unboosted (BCG group). Vaccination doses were identical for both species: BCG (5 × 10^6^ CFU/animal) and PLGA NPs (1 mg/animal).

Five weeks after the final immunization, animals were challenged via aerosol with *Mtb* H37Rv at a dose of 100 CFU/animal. Mice were euthanized at weeks 11, 15, 19, and 35, while guinea pigs were euthanized only at week 35 to assess the bacterial burden in the lungs and spleens.

Initial bacterial loads in the lungs and spleens of mice were comparable across all groups one-week post-challenge (Figure 5C,E). Statistically significant differences emerged as the infection progressed. While the bacterial burden increased steadily in unvaccinated control mice, BCG vaccination effectively controlled the infection. By the end of the observation period (week 35), mice that received a single BCG vaccination had markedly reduced mean bacterial loads in the lungs (33-fold lower) and spleen (102-fold lower) compared to PBS-treated controls. Similarly, in guinea pigs, BCG vaccination led to substantial reductions in bacterial burden in the lungs (292-fold) and spleens (142-fold) relative to unvaccinated animals (Figure 5D,F).

Interestingly, when using the PLGA-mock vaccine, which contains only immunostimulatory molecules, as a booster following primary BCG vaccination, we detected an even higher bacterial load in both animal models compared to the group that received BCG priming alone. As previously reviewed, PRR-mediated inflammatory responses in the absence of concomitant antigen presentation during *Mtb* infection may not have an exclusively beneficial effect on overall mycobacterial resistance [36,37]. The lowest bacterial loads were observed in the group vaccinated with BCG and boosted with the antigen-containing PLGA vaccine. This full vaccination regimen resulted in a further reduction in *Mtb* titers in the lungs and spleens of both mice (6.7- and 5.0-fold, respectively) and guinea pigs (6.9- and 11.3-fold, respectively) by week 35, compared to animals that received BCG alone.

### 3.6. PLGA-Based Multistage Vaccine Induces Protection in Post-Exposure Infection Model

**Figure 6:**
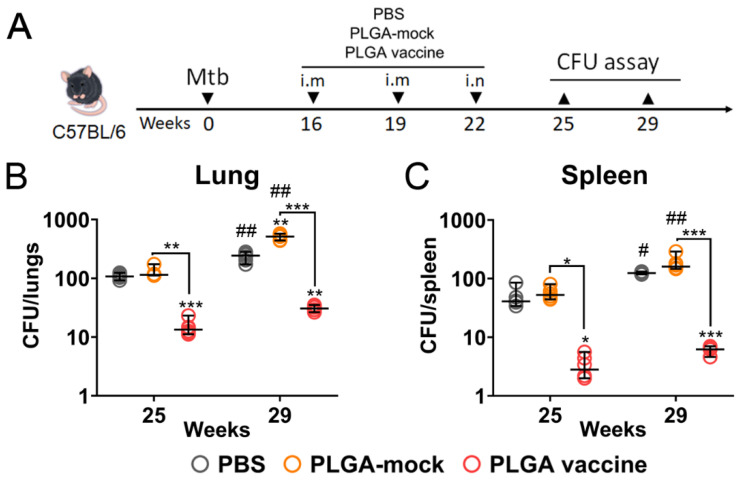
The protective efficacy of the PLGA-based multistage vaccine used in therapeutic regimen. (**A**) C57BL/6 mice were intraperitoneally infected with 10^4^ CFU/mouse *M. tuberculosis* H37Rv. 16 weeks later animals were randomly split to experimental groups receiving placebo (BCG), PLGA particles without fusion antigen (PLGA-mock) or PLGA-vaccine in three-dose immunization scheme with 3 weeks interval. Bacterial loads in mouse (**B**) lungs and (**C**) spleens were assessed at week 25 and 29. Dots represent individual data. Horizontal lines indicate the mean, and whiskers represent SD. Significant differences between different experimental groups are indicated by asterisks (two-way ANOVA coupled with Tukey’s multiple-comparisons test, * *p* < 0.05, ** *p* < 0.01, *** *p* < 0.005). Significant time-dependent differences between same experimental group are indicated by hashes (Mann–Whitney U test, # *p*< 0.05, ## *p* < 0.01).

Lastly, we examined the protective efficacy of the PLGA-based vaccine in a murine TB post-exposure model. Mice were intraperitoneally infected with *Mtb* (10^4^ CFU/animal) to mimic highly lethal disseminated form of tuberculosis [38,39]. Twelve weeks post-infection bacterial loads in the lungs and spleen in six randomly selected mice were quantified to confirm uniform infection. At 16 weeks post-infection, the remaining mice were split in experimental groups (12 mice/group) and received two IM injections followed with one IN immunization three weeks apart (Figure 6A).

Since the BCG vaccine is used exclusively as a prophylactic in clinical practice, mice were immunized with either the PLGA-based vaccine or a PLGA-mock (placebo) formulation. A control group of unvaccinated mice received PBS. The bacterial load in the lungs (Figure 6B) and spleens (Figure 6C) was assessed three and seven weeks post-immunization, with six mice per group. Consistent with a prophylactic regimen, the PLGA-mock formulation showed no beneficial effect; in fact, it resulted in a slight (×1.47 fold), though not statistically significant, increase in the bacterial burden in the organs. This indicates that innate immune reactions triggered by the immunostimulatory components of the vaccine formulation alone are insufficient to confer protection. In contrast, administration of the complete PLGA vaccine resulted in a substantial reduction in *Mtb* titers. At weeks 25 and 29, compared to unvaccinated animals, the bacterial loads in vaccinated mice decreased by 7.4- and 7.6-fold in the lungs and by 14.7- and 21.2-fold in the spleen, respectively. Importantly, while bacterial loads increased significantly over time in both the PBS- and PLGA-mock-treated groups (*p* < 0.01), the vaccinated mice showed no statistically significant progression of the *Mtb* infection.

## 4. Discussion

The development of a tuberculosis vaccine has entered a promising new phase, fueled by recent clinical data [40] and by new insights into mycobacterial persistence [41,42,43,44]. The discovery of new classes of *Mtb* antigens over the past decades has provided more options for vaccine antigen selection. Additionally, the failure of single-antigen approaches—most notably the disappointing results from the MVA85A vaccine trials—has reinforced the understanding that effective tuberculosis vaccines must address the complex, multistage nature of *Mtb* infection [20,45].

Indeed, today there are several examples indicating that multistage vaccines utilizing antigens from different stages of *M. tuberculosis* infection consistently outperform single-antigen formulations. The H56 vaccine (Ag85B-ESAT6-Rv2660c) and a nanoparticle formulation containing Ag85A, ESAT-6, CFP10, Rv2660c, and TB10.4 antigens have shown superior protection compared to single-antigen vaccines in mice [46,47], while the multistage vaccine LT69 (ESAT6-Ag85B-MPT64-Mtb8.4-HspX) provided long-term animal protection approaching that of BCG [48]. The CMFO (Rv2875-Rv3044-Rv2073c-Rv0577) multistage vaccine achieved sterile immunity in BCG-primed mice, a remarkable outcome presumably attributed to its comprehensive antigenic coverage [49].

Guided by this logic, we developed a novel multi-antigenic PLGA-based subunit vaccine. It incorporates antigens from both the active (Ag85A, ESAT6, CFP10) and latency-associated (Rv2660c, Rv1813c) stages of mycobacterial infection. While the protective efficacy of the individual antigens is well-established, their specific combination and formulation are novel.

Three of the five antigens used (Ag85A, ESAT6, and CFP10) are currently being evaluated in the GamTBVac vaccine, which has been shown to be safe and immunogenic in BCG-primed individuals in a Phase 2 study and is now in an ongoing Phase 3 trial [26]. The selection of Rv1813c was based on its association with latency and its ability to induce strong immune responses. Clinical trials have demonstrated that ID93 + GLA-SE is safe and well-tolerated, inducing robust antigen-specific serum antibody responses and Th1-type cellular immune responses [50]. The inclusion of Rv2660c in a PLGA vaccine was justified by promising Phase 1/2 clinical trial results for the H56:IC31 candidate, which demonstrated safety and immunogenicity in tuberculosis patients [51]. Although the three vaccine antigens—Ag85B, ESAT-6, and Rv2660c—are all implicated in tuberculosis recurrence and relapse, a subsequent investigation revealed that administering H56:IC31 at the completion of pulmonary tuberculosis treatment did not reduce the risk of recurrent disease [52]. This result suggests that the problem may not be connected to the antigens themselves, but rather may lie with an insufficient antigen repertoire and/or inadequate resulting vaccine immunogenicity, which failed to generate a protective immune response.

To address this, the immunogenicity of the chosen vaccine’s broad antigen composition was enhanced using an original synergistic complex adjuvant containing MPLA (a TLR4 agonist) and MDP (a NOD2 agonist). This strategy is based on a synergistic model of innate immune activation, which has been validated by recent studies. Building on our initial findings that demonstrated TLR4 and NOD2 synergy in shaping adaptive immune responses to a model antigen (ovalbumin) [29], this approach has also shown promise as an effective immunotherapy for enhancing the efficacy of TB drugs [53].

Finally, to co-immobilize the active vaccine components, we employed a biocompatible and non-toxic PLGA carrier [54]. Produced using modern microfluidic techniques, this PLGA-based nanoparticle platform offers a scalable and cost-effective delivery system. It enables the incorporation of multiple *Mtb* proteins and immunostimulatory molecules with diverse physicochemical properties into a single vaccine particle. Furthermore, encapsulating antigens in PLGA nanoparticles has been demonstrated to enhance the magnitude and breadth of the immune response. For example, Szachniewicz et al. encapsulated an Ag85B-ESAT6-Rv2034 fusion protein in PLGA particles and reported robust multifunctional CD4^+^ and CD8^+^ T-cell responses, along with measurable control of bacterial load in mice [55].

The physicochemical characteristics of PLGA nanoparticles, particularly their size, significantly influence vaccine immunogenicity. Size governs cellular uptake mechanisms, intracellular trafficking, and the type of immune response elicited. This size-dependence has been extensively documented, with optimal dimensions—generally between 20 and 200 nm—considered essential for promoting a Th1-biased cellular immune response [56,57]. Supporting this, PLGA nanoparticles of 200–300 nm loaded with Ag85B and ESAT-6 antigens have demonstrated promising results, inducing robust populations of IFN-γ-producing CD4+ T cells and cytotoxic CD8+ T cells. Both of these effector cell types are vital for controlling intracellular *M. tuberculosis* infection [23].

The present study was aimed to investigate immunogenicity of a novel design of nanoscale (≈150nm) multistage fusion vaccine co-loaded with complex adjuvant and its protectivity in prophylactic (with cross-species validation) and therapeutic regimens.

Vaccine’s immunogenicity in mice was assessed via a mixed-route immunization strategy (two intramuscular immunizations followed by one intranasal) shown to be effective for mounting robust systemic as well as local T-cell-mediated response in the lungs [58].

The observed vaccine-induced Th1/Th17 profile in secondary lymphoid organs is consistent with growing evidence that, alongside the well-established role of IFN-γ-producing Th1 cells [59], IL-17 is a key mediator of vaccine protection [60,61]. Specifically, Counoupas et al. demonstrated that blocking IL-17 abrogated the protection conferred by an Advax-formulated CysVac2 mucosal vaccine, linking this cytokine to both phagocyte recruitment and the optimal priming of secondary T-cell responses [62].

Vaccines that induce both T-cell and B-cell responses are considered preferable for achieving significant protection in experimental TB models [63]. The high titers of IgA detected in the BAL and long-lasting IgG in the serum of vaccinated mice likely contribute to protection against TB [64,65]. There is emerging evidence supporting the importance of these antibodies as correlates of protection and potential targets for vaccine development. Recent studies have demonstrated that antibodies specific to *Mycobacterium obuense* sonicate (MOS) or BCG correlate with protection against TB in humans and non-human primates [66,67]. The importance of mucosal IgA in TB protection was further underscored by recent data indicating that household contacts who remained QuantiFERON-negative despite exposure to active TB cases had higher serum heparin-binding hemagglutinin adhesin (HBHA)-specific IgA responses than patients with active TB, suggesting a protective role for IgA in preventing disease progression [68].

Since the prepared PLGA-based vaccine induced durable humoral and balanced Th1/Th17 responses, we evaluated its protective efficacy on two fundamentally different infection models to assess vaccine efficacy across the spectrum of tuberculosis vaccination scenarios.

In the pre-exposure model, which represents the traditional prophylactic vaccination approach, BCG-primed animals were boosted by subsequent intramuscular or intranasal immunization with a PLGA-based vaccine, followed by an aerosol challenge. In both animal models, the bacterial burden in the lungs and spleen demonstrated a statistically significant (5–11-fold) reduction compared to unvaccinated controls, consistent with the established efficacy of BCG in animal models [69]. Furthermore, boosting with the multistage vaccine provided an additional statistically significant reduction in lung and spleen bacterial loads, demonstrating the synergistic protective effect of a heterologous prime-boost strategy. This finding aligns with recent clinical evidence suggesting that BCG-primed individuals may benefit from heterologous boosting with protein subunit vaccines [70].

The post-exposure model, established via intraperitoneal *M. tuberculosis* infection, does not replicate the natural primary pathogenesis of pulmonary tuberculosis. However, it is considered to mimic other aspects of human *M. tuberculosis* infection. Extrapulmonary tuberculosis—defined by WHO criteria as infection affecting tissues and organs outside the pulmonary parenchyma [71,72,73]—accounts for approximately 15% of all TB cases [74]. Published literature suggests that extrapulmonary TB may represent either an advanced stage of initial pulmonary infection or an alternative pathogenesis scenario [75]. In this context, experimental studies propose that intraperitoneal *M. tuberculosis* infection can model certain features of such an extrapulmonary stage [76], potentially even during slowly progressive primary tuberculosis [38]. Accordingly, in this study we aimed to evaluate the protective efficacy of a PLGA vaccine in this model of disseminated extrapulmonary *Mtb* infection. Triple therapeutic PLGA vaccination resulted in a statistically significant (7–20-fold) reduction in bacterial burden in both the lungs and spleen and prevented disease progression over time. In contrast, the antigen-free vaccine formulation (PLGA-mock) slightly, though not statistically significantly, promoted infection.

Rodent models are invaluable in infection research, yet they possess inherent limitations for translating findings to humans. Tuberculosis research exemplifies this challenge. Specifically, mouse models fail to develop key pathological hallmarks seen in humans, including caseating granulomas, cavitary lesions, and the full spectrum of latent infection [77,78]. Therefore, extrapolating vaccine efficacy from rodent data must account for significant species-specific immune differences. To establish a more robust and translational foundation, this study evaluated a PLGA-based multistage vaccine using a comparative approach across multiple species and modes of infection. This methodology generates a comprehensive profile of protective immunity, strengthening confidence in its potential for human application.

Progression to non-human primate (NHP) models is a critical subsequent step in preclinical vaccine research. NHPs closely mirror human immunology and have extended lifespans, making them uniquely suited for studying long-term immune responses relevant to humans [79,80]. These include defining the durability of immune protection and evaluating the necessity for booster vaccinations. Furthermore, NHP models enable a detailed assessment of mucosal immunity in the respiratory tract, which is paramount for an airborne disease like TB caused by *Mtb*.

## 5. Conclusions

In summary, this study demonstrates that a rationally designed multistage subunit vaccine delivered via PLGA nanoparticles with complex immunoadjuvants can provide protection against tuberculosis in both prophylactic and therapeutic settings. Our results support the continued development of multistage vaccines targeting both active and latent phases of tuberculosis infection and validate the potential of nanoparticle-based delivery systems with synergistic adjuvant combinations. These findings contribute to the growing body of evidence supporting next-generation tuberculosis vaccines that may finally provide the tools necessary to achieve global tuberculosis control goals.

## Data Availability

Data are available from the authors upon reasonable request.

## Supplementary Materials

The following supporting information can be downloaded at https://www.mdpi.com/article/10.3390/vaccines14010005/s1. Figure S1: Peptide sequence coverage map of the recombinant ESAT6-CFP10-Ag85A-Rv2660c-Rv1813c fusion protein, obtained by LC–MS/MS analysis. The map displays the full amino acid sequence of the protein (in black). Regions corresponding to theoretical tryptic peptides are marked in green, while gray shading highlights the experimentally confirmed peptides identified by LC–MS/MS. The blue lines below the sequence represent the identified peptides, aligned to their theoretical positions. The coverage map shows nearly complete sequence coverage (≈80%), with dense, overlapping peptides across most regions. The central and C-terminal regions, in particular, exhibited extensive and redundant coverage, confirming the integrity of the expressed sequence. Overall, over 300 unique tryptic peptides were identified, providing strong fragment-ion evidence for the majority of residues; Figure S2: Stability of the prepared PLGA vaccine particles was assessed in 1x PBS at room temperature over 28 days. (A) Changes in particle size, count, and antigen content on days 0, 2, 7, and 28. (B) Representative nanoparticle tracking analysis (NTA) results for the PLGA vaccine particles on days 0, 2, 7, and 28.
