# Supplementary material for: Novel Multistage Subunit Mycobacterium tuberculosis Nanoparticle Vaccine Confers Protection Against Experimental Infection in Prophylactic and Therapeutic Regimens
Source: Vaccines (Basel). 2025 Dec 19;14(1):5. doi: 10.3390/vaccines14010005 (PMC12846566; doi:10.3390/vaccines14010005)
Supplement: Supplementary file 1 [file vaccines-14-00005-s001.zip › vaccines-3999326-supplementary.pdf]

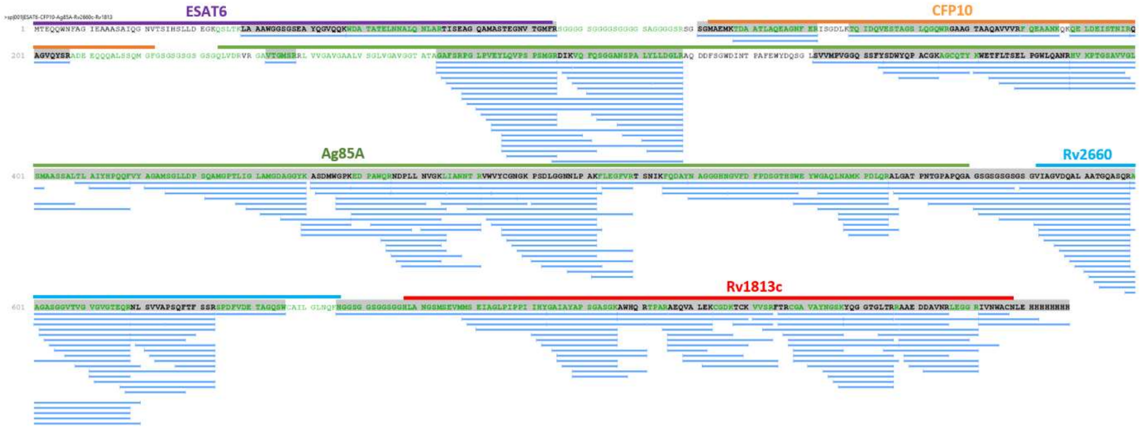

Figure S1: Peptide sequence coverage map of the recombinant ESAT6-CFP10-Ag85A-Rv2660c-Rv1813c fusion protein, obtained by LC–MS/MS analysis. The map displays the full amino acid sequence of the protein (in black). Regions corresponding to theoretical tryptic peptides are marked in green, while gray shading highlights the experimentally confirmed peptides identified by LC–MS/MS. The blue lines below the sequence represent the identified peptides, aligned to their theoretical positions. The coverage map shows nearly complete sequence coverage (≈80%), with dense, overlapping peptides across most regions. The central and C-terminal regions, in particular, exhibited extensive and redundant coverage, confirming the integrity of the expressed sequence. Overall, over 300 unique tryptic peptides were identified, providing strong fragment-ion evidence for the majority of residues

A

	Day 0	Day 2	Day 7	Day 28
Average Number of Particles per Dose ± SD, 10^8	2.86 ± 0.22	3.05 ± 0.13	2.86 ± 0.06	2.51 ± 0.11
Diameter, nm Mean ± SD	151.4 ± 36.5	152.8 ± 45.4	151.2 ± 37.2	153.9 ± 41.0
Total antigen (µg per mg)	5.28	5.19	5.25	5.46

B

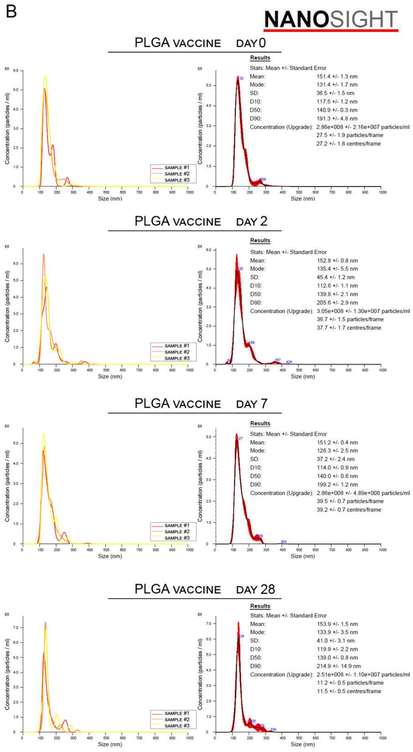

Figure S2: Stability of the prepared PLGA vaccine particles was assessed in 1x PBS at room temperature over 28 days. (A) Changes in particle size, count, and antigen content on days 0, 2, 7, and 28. (B) Representative nanoparticle tracking analysis (NTA) results for the PLGA vaccine particles on days 0, 2, 7, and 28.
